# New ridge parameter estimators for the quasi-Poisson ridge regression model

**DOI:** 10.1038/s41598-023-50085-5

**Published:** 2024-04-11

**Authors:** Aamir Shahzad, Muhammad Amin, Walid Emam, Muhammad Faisal

**Affiliations:** 1 Department of Statistics, University of Sargodha, Sargodha, Pakistan; 2 Department of Statistics and Operations Research, Faculty of Science, King Saud University, P.O. Box 2455, 11451 Riyadh, Saudi Arabia; 3 Faculty of Health Studies, University of Bradford, Bradford, UK

**Keywords:** Applied mathematics, Statistics

## Abstract

The quasi-Poisson regression model is used for count data and is preferred over the Poisson regression model in the case of over-dispersed count data. The quasi-likelihood estimator is used to estimate the regression coefficients of the quasi-Poisson regression model. The quasi-likelihood estimator gives sub-optimal estimates if regressors are highly correlated—multicollinearity issue. Biased estimation methods are often used to overcome the multicollinearity issue in the regression model. In this study, we explore the ridge estimator for the quasi-Poisson regression model to mitigate the multicollinearity issue. Furthermore, we propose various ridge parameter estimators for this model. We derive the theoretical properties of the ridge estimator and compare its performance with the quasi-likelihood estimator in terms of matrix and scalar mean squared error. We further compared the proposed estimator numerically through a Monte Carlo simulation study and a real-life application. We found that both the simulation and application results show the superiority of the ridge estimator, particularly with the best ridge parameter estimator, over the quasi-likelihood estimator in the presence of multicollinearity issue.

## Introduction

Regression analysis is widely used for predicting or identifying the factors associated with the response variable. Several types of regression models are developed that depend on the distribution of the response variable, and the type of relationship (linear vs non-linear) to measure the average relationship between the response variable and one or more explanatory variable variables such as the generalized linear model (GLM)^1^ and some other models. The GLM has various applications in science, engineering, business and some others^2,3^.

The count models are used to examine the factors that influence the response variable which is in positive integers such as 0, 1, 2, 3, 4…, etc.^4,5^. Several count regression type models are developed such as the Poisson regression model (PRM), Quasi-Poisson regression model (QPRM), negative binomial regression model (NBRM), bell regression model (BRM) and Conway Maxwell Poisson regression model (CMPRM). These models are used in various situations. The PRM^6^ is used when the response variable has a Poisson distribution with identical average and dispersion^7^.

In real-life datasets, the assumption of equal mean and variance, as postulated by PRM, often is violated. Over-dispersion occurs when the variance of the response variable exceeds its mean. The NBRM is used to handle over-dispersion^8^. However, NBRM requires a large number of samples and the response variable must have positive numbers. This model is commonly applied to count data and over-dispersion scenarios^9^. Conway and Maxwell introduced the Conway–Maxwell PRM (CMPRM) in 1962, adept at addressing both over and under-dispersion in count data^10,11^. Additionally, the QPRM is a generalization of the PRM and is commonly used for modeling an over-dispersed count variable^12^. The QPRM is an alternative to the NBRM^13^ and is recommended when the variance is a linear function of the mean^13^.

The quasi-likelihood estimator (QLE) is used to estimate the regression coefficients of the QPRM^14^. However, it produces inefficient estimates when the regressors are highly correlated known as multicollinearity. This term was used by Frisch^15^ for the first time in the linear regression model. When multicollinearity exists in a QPRM, the QLE produces large variances, leading to incorrectly signed regression coefficients and wider confidence intervals. This issue further results in inflated standard errors of regression coefficients, potentially leading to inaccurate outcomes^16^.

Biased estimation methods are commonly used to mitigate the impact of multicollinearity. Several biased estimation methods have been developed to diminish the impact of multicollinearity in producing more reliable estimates^17–19^. The ridge estimator (RE) is a popular method to deal with the issue of multicollinearity in the linear regression model^20^. It shrinks the coefficients of the model and diminishes the impact of multicollinearity. A key component of the RE is the biasing parameter *k* crucial for optimizing regression coefficients. Several studies found the optimal *k* value by minimizing the Mean Squared Error (MSE) to enhance the estimator's performance^20–28^. Various studies determine the optimal value of biasing parameters in RE with different probability-distributed models^7,18,25,29–33^.

Various studies proposed the REs to diminish the impact of multicollinearity in a count regression model such as, RE for Poisson ridge regression^7,18,34,35^, RE for the NBRM^30,36^, RE for Bell regression model^32^ and more recently, the RE for the CMPRM^37^.

It is evident from the literature that several studies proposed REs and some new biasing parameters for the linear regression model, count regression model and GLMs. Furthermore, these studies attempted to find the best ridge parameter estimators (RPEs) of the above models. However, no study has investigated RE for the QPRM. In this study, we aimed to investigate the RE for the QPRM to deal with multicollinearity and over-dispersion and compare the RE and the QLE in the QPRM. Furthermore, we aim to introduce diverse RPEs tailored for the QPRM's ridge estimator and assess their performance to identify the most effective one.

The rest of the paper is organized as follows: section “Methodology” will cover the structure, estimation, and properties of the QPRM. Additionally, we will introduce the RPEs in this section. The simulation study’s setting, its results and application will be discussed in section “Numerical evaluation”. Section “Application: apprentice migration dataset” will discuss the results of real-life application. The concluding remarks will be given at the end of this paper.

## Methodology

### The QPRM

The QPRM is used in the situation, where the dependent variables, ${y}_{i}$ is in counting form, follows the Poisson distribution and its variance is greater than its mean. The quasi-Poisson (QP) distribution is considered for analyzing the over-dispersion condition in the datasets. For detailed discussion and properties, see Efron^38^ and Istiana et al.^39^. The QP distribution has a relation with the Poisson distribution when the dispersion parameter is equal to 1. Let us consider *Y,* which represents the response variable selected from the QP distribution with parameters $\mu {\text{ and }} \gamma$. The mean and variance of the QP are respectively given by

1 $${\text{E}}({\text{Y}}) =\upmu$$

2 $${\text{Var}}\left({\text{Y}}\right)= \gamma\upmu ,$$

where µ > 0, and $\gamma$ is an over-dispersion parameter. The close relationship between the expectation and variance shows that variance is a function of its average. The QPRM is characterized by the first two moments (mean and variance) as discussed by Wedderburn^12^, but Efron^38^ and Gelfand and Dalal^40^ showed how to create a distribution for this model; however, it requires re-parameterization. Estimation often proceeds from the first two moments and estimating equations^14^. The likelihood function for the QPRM (quasi-likelihood) does not require a specific probability density function to estimate regression parameters except for the response variable assumption^41^. The formation of the QL function begins in the same way as the usual likelihood function. The QLE is obtained by minimizing the Quasi-log likelihood function that is given by Wakefield^42^

3 $$l\left({y}_{i}, {\mu }_{i}\right)=\frac{1}{\gamma }\left({y}_{i}log {\mu }_{i}-{\mu }_{i}\right)$$

Now differentiate (3) w.r.t. ${\mu }_{i}$, and equating to zero, we have the Quasi-score function

4 $${U}_{i}=\frac{{y}_{i}- {\mu }_{i}}{\gamma {\mu }_{i}},$$

where $\mu =exp\left(X\beta \right)$. Here $X$ is a covariates matrix of order $n \times (p + 1)$ and $\beta$ is the column vector of regression coefficients of order $(p + 1) \times 1$.

As Eq. (4) is non-linear in $\beta$, so, the regression parameters of the QP model can be calculated using iterative reweighted least square (IWLS). The equation used in calculating the IWLS with $t+1$ iterations can be written as^13^

5 $${\beta }^{\left[t+1\right]}={\left({X}^{\prime}{\widehat{{\text{W}}}}^{\left[t\right]}X\right)}^{-1}{X}^{\prime}{W}^{\left[t\right]}{m}^{\left[t\right],}$$

where $${\widehat{{\text{W}}}}_{i}^{\left[t\right]}=\frac{1}{V({\mu }_{i}^{\left[t\right]})}{\left(\frac{\partial {\mu }_{i}^{\left[t\right]}}{\partial {\eta }_{i}^{\left[t\right]}}\right)}^{2},$$ $${m}_{i}^{\left[t\right]}={\eta }_{i}^{\left[t\right]}+\frac{\left({y}_{i}-{\mu }_{i}^{\left[t\right]}\right)}{\frac{\partial {g}^{-1}\left({\eta }_{i}^{\left[t\right]}\right)}{\partial {\eta }_{i}^{\left[t\right]}}}$$, $${\eta }_{i}^{\left[t\right]}={x}_{i}^{\prime}{\beta }^{\left[t\right]}$$,

$${\mu }_{i}^{\left[t\right]}={g}^{-1}\left({\eta }_{i}^{\left[t\right]}\right)={g}^{-1}\left({x}_{i}^{\prime}{\beta }^{\left[t\right]}\right),$$

where ${\mu }_{i}={\text{exp}}\left({\eta }_{i}\right)$. So, ${m}_{i}={\text{log}}\left({\mu }_{i}\right)+\frac{{y}_{i}-{\mu }_{i}}{{\mu }_{i}}$. The QLE of $\beta$ at the final iteration is defined as

6 $${\widehat{\beta }}_{QLE}={\left(F\right)}^{-1}{X}^{\prime}\widehat{W}m,$$

where $F={{\text{X}}}^{{{\prime}}}\widehat{{\text{W}}}{\text{X and }}\widehat{\text{ W}}={\text{diag}}\left(\frac{1}{V(\widehat{\mu })}{\left(\frac{\partial \widehat{\mu }}{\partial \eta }\right)}^{2} \right).$ The QLE is normally distributed with a covariance matrix that corresponds to the inverse of the matrix of the second derivatives:

7 $$Cov\left({\beta }_{QLE}\right)={\left[E\left(-\frac{{\partial }^{2}l({\text{X}};\upbeta )}{\partial \beta \partial {\beta }^{\prime}}\right)\right]}^{-1}={\gamma \left({\text{F}}\right)}^{-1}.$$

Furthermore, the MSE of the ${\widehat{\beta }}_{QLE}$ is given as

8 $$MSE\left({\widehat{\beta }}_{QLE}\right)=E{\left({\beta }_{QLE}-\beta \right)}^{\prime}\left({\beta }_{QLE}-\beta \right)=\gamma {\left({\text{F}}\right)}^{-1}$$

By applying the trace on both sides of Eq. (8), we have

9 $$MSE\left({\widehat{\beta }}_{QLE}\right)=\gamma {\text{tr}}{\left({\text{F}}\right)}^{-1}=\gamma \sum_{j=1}^{p+1}\frac{1}{{\lambda }_{j}},$$

where ${\lambda }_{j}$ is the *j*th eigenvalue of the matrix $F$. When the explanatory variables in the QPRM are highly correlated, then the weighted matrix of cross-products of $F,$ is ill-conditioned, and the QLE gives inefficient results with larger variances. In this condition, it is difficult to interpret the estimated coefficients since the vector of parameters is on average too large.

### The quasi Poisson ridge regression estimator

When the multicollinearity is present among the explanatory variables in the QPRM, then the QLE does not perform efficiently, it gives a large variance of estimated coefficients. To minimize the effects of multicollinearity, the RR estimation method was introduced by Hoerl and Kennard^20^. In this study, we are proposing the quasi-Poisson ridge regression estimator (QPRRE) applied to the count and over-dispersed data that minimizes the effects of multicollinearity. So, the QPRRE is defined by

10 $${\widehat{\beta }}_{k}={\left(F+k{I}_{p}\right)}^{-1}F{\widehat{\beta }}_{QLE},$$

where $F={{\text{X}}}^{{{\prime}}}\widehat{{\text{W}}}{\text{X}}$ and *k* is the biasing parameter and ${I}_{p+1}$ is the identity matrix with order $(p+1)\times (p+1)$. The bias, the covariance and the matrix MSE (MMSE) of the ${\widehat{\beta }}_{k}$ can be derived as follows

11 $$Bias \left({\widehat{\beta }}_{k}\right)=-kT{\Lambda }_{k}^{-1}\beta .$$

12 $$Cov\left({\widehat{\beta }}_{k}\right)=\widehat{\gamma }T{\Lambda }_{k}^{-1}\Lambda {\Lambda }_{k}^{-1} {T}^{\prime}.$$

$$MMSE\left({\widehat{\beta }}_{k}\right)=Cov\left({\widehat{\beta }}_{k}\right)+Bias \left({\widehat{\beta }}_{k}\right)Bias {\left({\widehat{\beta }}_{k}\right)}^{\prime}$$

13 $$MMSE\left({\widehat{\beta }}_{k}\right)=\widehat{\gamma }T{\Lambda }_{k}^{-1}\Lambda {\Lambda }_{k}^{-1} {T}^{\prime}+{k}^{2}T{\Lambda }_{k}^{-1}\beta {\beta }{\prime}{\Lambda }_{k}^{-1}{T}^{\prime},$$

where ${\Lambda }_{k}=diag({\lambda }_{1}+k,{\lambda }_{2}+k,\ldots,{\lambda }_{p+1}+k)$ and $\Lambda =diag({\lambda }_{1},{\lambda }_{2}, {\lambda }_{3}, \ldots,{\lambda }_{p+1})=T\left(F\right){T}^{\prime}$, where the orthogonal matrix $T$ has eigenvectors of $F$. Finally, the scalar MSE of the QPRRE can be estimated by applying trace on Eq. (13), which can be defined as

14 $$\begin{aligned} MSE\left({\widehat{\beta }}_{k}\right)&=tr\left\{MMSE\left({\widehat{\beta }}_{k}\right)\right\} \\ MSE\left({\widehat{\beta }}_{k}\right)&=\widehat{\gamma }{\sum }_{ j=1}^{p+1}\frac{{\lambda }_{j}}{{\left({\lambda }_{j}+k\right)}^{2}}+{k}^{2}{\sum }_{j=1}^{p+1}\frac{{{\alpha }_{j}}^{2}}{{\left({\lambda }_{j}+k\right)}^{2}}={M}_{1}\left(k\right)+{M}_{2}\left(k\right), \end{aligned}$$

where ${\alpha }_{j}={{T}^{\prime}\widehat{\beta }}_{QLE}$ and ${\lambda }_{j}$ is the *j*th eigenvalue of the *F* matrix.

### The superiority of the QPRRE to the QLE

To explore the superiority of the RR estimator over others, Hoerl and Kennard^20^ proposed the statements for the properties of the MSE of the RR estimators in the LRM. Here, we will prove that the theorems also hold for the QPRM and according to these theorems, we will explore the supremacy of the QPRRE over the QLE.

#### Theorem 3.1

*The variance* ${M}_{1}\left(k\right)$ *and squared bias* ${M}_{2}\left(k\right)$ *are respectively continues, monotonically decreasing and increasing functions of* *k*, *since* $k>0$ *and* ${\lambda }_{j}>0$.

#### *Proof*

The 1st derivative of ${M}_{1}\left(k\right)$ and ${M}_{2}\left(k\right)$ from Eq. (14) concerning *k* are*.*

15 $${M}_{1}^{\prime}=-2\widehat{\gamma }{\sum }_{ j=1}^{p+1}\frac{{\lambda }_{j}}{{\left({\lambda }_{j}+k\right)}^{3}}$$

and

16 $${M}_{2}^{\prime}=2k{\sum }_{j=1}^{p+1}\frac{{{\alpha }_{j}}^{2}{\lambda }_{j}}{{\left({\lambda }_{j}+k\right)}^{3}}$$

The $\widehat{\gamma },k,{\lambda }_{j}$ and ${{\alpha }_{j}}^{2}$ are positive, Eq. (15) shows that the ${M}_{1}\left(k\right)$ is a continuous and monotonically decreasing function of *k*, since $k>0$ and ${\lambda }_{j}>0$. Equation (16) shows that the ${M}_{2}\left(k\right)$ is a continuous and monotonically increasing function of *k.*

#### Theorem 3.2

*There always exists a* $k>0$ *and* *the* $MSE\left({\widehat{\beta }}_{k}\right)<MSE\left({\widehat{\beta }}_{QLE}\right).$

#### *Proof*

The 1st derivative of Eq. (14) concerning *k* is given by

17 $$\begin{aligned} \frac{dMSE\left({\widehat{\beta }}_{k}\right)}{dk}&=-2\widehat{\gamma }{\sum }_{ j=1}^{p+1}\frac{{\lambda }_{j}}{{\left({\lambda }_{j}+k\right)}^{3}}+2k{\sum }_{j=1}^{p+1}\frac{{{\alpha }_{j}}^{2}{\lambda }_{j}}{{\left({\lambda }_{j}+k\right)}^{3}}\\ &=-2\sum_{j=1}^{p+1}\frac{{\lambda }_{j}\left(\widehat{\gamma }-k{{\alpha }_{j}}^{2}\right)}{{\left({\lambda }_{j}+k\right)}^{3}} \end{aligned}$$

Equation (17), clearly shows that the sufficient condition for $\frac{dMSE\left({\widehat{\beta }}_{k}\right)}{dk}$ to be less than zero is $\frac{\widehat{\gamma }}{{{\alpha }_{j}}_{max}^{2}}<0$.

### Selection of the biasing parameters

The RR estimator is based on the RPE which has a main role in its estimation. To minimize the effects of high correlation among the explanatory variables, the optimal value of the shrinkage parameter (*k*) is the main concern of the RR. The RPEs for different regression models have been suggested by many investigators and find the optimal biasing parameter^20,24,31,37,43,44^. Firstly, Hoerl and Kennard^20^ presented the ridge estimation method to mitigate the effect of a high degree of correlation for the LRM. This estimator was also used for the gamma regression model (GRM)^44^, and for the CMPRM^37^. For the QPRRE, it is defined as

18 $${k}_{1}=\frac{\widehat{\gamma }}{\sum_{j=1}^{p+1}{\widehat{\alpha }}_{j}^{2}}$$

where $\widehat{\gamma }$ is the estimated dispersion parameter.

Hoerl et al.^24^ proposed the shrinkage parameter estimator for the RR in the LRM and we are adapting this estimator for the QPRRE as

19 $${k}_{2}=\frac{\left(p+1\right) \widehat{\gamma }}{\sum_{j=1}^{p+1}{\widehat{\alpha }}_{j}^{2}}$$

Amin et al.^16,44^ developed an RPE for the inverse Gaussian ridge regression (IGRR)

20 $${k}_{3}={\text{max}}\left({k}_{j}\right),\quad \text{where }{ k}_{j}=\frac{\widehat{\gamma }}{{\widehat{\alpha }}_{j}^{2}}.$$

Akram et al.^45^ proposed the following RPEs of the GRM’s RR.

21 $${k}_{4}=\frac{\widehat{\gamma }}{p+1}\sum_{j=1}^{p+1}\left(\frac{1 }{2{\widehat{\alpha }}_{j}^{2}+max\left(\frac{\widehat{\gamma }}{{\widehat{\lambda }}_{j}}\right)}\right)$$

22 $${k}_{5}=median\left(\frac{\widehat{\gamma }}{2{\widehat{\alpha }}_{min}^{2}+\left(\frac{\widehat{\gamma }}{{\widehat{\lambda }}_{j}}\right)}\right)$$

23 $${k}_{6}=\frac{\widehat{\gamma }}{p+1}\sum_{j=1}^{p+1}\left[\frac{1}{2{\widehat{\alpha }}_{j}^{2}+\left(\frac{\widehat{\gamma }}{{\widehat{\lambda }}_{j}}\right)}\right]$$

24 $${k}_{7}=median\left(\frac{\widehat{\gamma }}{2{\widehat{\alpha }}_{j}^{2}+\left(\frac{\widehat{\gamma }}{{\widehat{\lambda }}_{j}}\right)}\right)$$

25 $${k}_{8}=\left[\frac{1}{\widehat{\gamma }}\left\{max\left[\frac{\widehat{\gamma }}{2{\widehat{\alpha }}_{j}^{2}+max\left(\frac{\widehat{\gamma }}{{\widehat{\lambda }}_{j}}\right)}\right]\right\}\right]$$

26 $${k}_{9}=\prod_{j=1}^{p+1}{\left(\frac{\widehat{\gamma }}{2{\widehat{\alpha }}_{j}^{2}+\left(\frac{\widehat{\varnothing }}{{\widehat{\lambda }}_{j}}\right)}\right)}^{\frac{1}{p+1}}$$

Given above stated studies, we proposed the following RPEs for the QPRRE as

27 $${k}_{10}={\text{min}}\left[\frac{\left(\widehat{\gamma }{\widehat{\lambda }}_{j}\right)}{{\widehat{\alpha }}_{j}^{2}}\right],$$

28 $${k}_{11}=\sum_{j=1}^{p+1}\left[\left(\frac{\widehat{\gamma }}{{\widehat{\alpha }}_{j}^{2}}\right)\left(1+\left(1+{\left(\frac{\widehat{\gamma }}{{\widehat{\alpha }}_{j}^{2}}\right)}^{2}\right)\right)\right],$$

29 $${k}_{12}=\sum_{j=1}^{p+1}\left(\frac{{\widehat{\lambda }}_{j}\widehat{\gamma }}{\widehat{\gamma }+{\widehat{\lambda }}_{j}{\widehat{\alpha }}_{j}^{2}}\right),$$

30 $${k}_{13}=\frac{1}{max\left({\widehat{\alpha }}_{j}^{2}\right)},$$

31 $${k}_{14}=\sum_{j=1}^{p+1}\left(\frac{1}{{\widehat{\alpha }}_{j}^{2}}\right)$$

32 $${k}_{15}=\sum_{j=1}^{p+1}\left[\frac{{\widehat{\lambda }}_{j}}{(1+2{\widehat{\lambda }}_{j}{\widehat{\alpha }}_{j}^{2})}\right]$$

33 $${k}_{16}=(p+1)\sum_{j=1}^{p+1}\left[\frac{\widehat{\gamma }}{2{\widehat{\alpha }}_{j}^{2}+\left(\frac{\widehat{\gamma }}{{\widehat{\lambda }}_{j}}\right)}\right]$$

## Numerical evaluation

In this section, a Monte Carlo simulation will be conducted to examine the performance of the RPEs in the QPRRE along with the QLE.

### Simulation layout

In the QPRM, the response variable is generated from the quasi-Poisson distribution $({\mu }_{i}, \gamma )$, where

34 $${\mu }_{i}={\text{exp}}\left({\beta }_{0}+{\beta }_{1}{x}_{ij}+{\beta }_{2}{x}_{ij}+\cdots +{\beta }_{p}{x}_{ip}\right), i={1,\,2},\ldots,n,$$

where ${\beta }_{p}$ shows the regression parameters of the QPRM. These parameters are selected under the condition of $\sum_{j=1}^{p+1}{\beta }_{j}^{2}=1$. And, the following formula is used to generate the correlated regressors.

35 $${x}_{ij}={\left(1-\rho \right)}^{1/2}{z}_{ij}+\rho {z}_{i\left(j+1\right)},\quad i={1,\,2},\ldots n;\;\; j={1,2},\ldots ,p,$$

where ${\rho }^{2}$ shows the correlation between the regressors and ${z}_{ij}$ are the independent standard normal pseudo-random numbers. We consider different values of $\rho$ corresponding to 0.80,0.90,0.95 and 0.99. We also consider different values of $n,p, \gamma$. Here, $n$ represents the sample size that is assumed to be 25, 50, 100, 150, 200 for *p* = 3, 6 and *n* = 50, 100, 150, 200 for *p* = 12, $p$ shows the number of regressors that are assumed to be 3, 6, 12 and $\gamma$ indicates the dispersion parameter that is taken to be 2, 4, 6. This simulation is replicated 2000 times with the different combinations of $n, p, \gamma , \rho$. To check the dominance of our proposed ridge estimator with different RPEs, we use the MSE as the performance evaluation method defined by

36 $$MSE\left(\widehat{\beta }\right)=\frac{\sum_{i=1}^{V}{\left({\widehat{\beta }}_{i}-\beta \right)}^{\prime}\left({\widehat{\beta }}_{i}-\beta \right)}{V},$$

where $V$ represents the number of replications and $\left({\widehat{\beta }}_{i}-\beta \right)$ shows the difference between the true parameter and predicted vectors of the proposed estimator and QLE at *i*th replication. The R programming language is used for all calculations related to our study.

### Simulation results and discussions

The estimated MSEs of the QPRREs are given in Tables 1, 2, 3, 4, 5, 6, 7, 8, 9, 10, 11, 12, 13, 14, 15, 16, 17, 18. We evaluate the performance of different RPEs for the QPRRE as compared to the QLE in the presence of multicollinearity, the sample size, dispersion and the number of regressors.

**Table 1:** Estimated MSEs for $\gamma =2$, *p* = 3 and $n=25$. Bold value indicates minimum MSE.

$\rho$	0.8	0.9	0.95	0.99
MSE (MLE)	26.92	55.04	109.02	553.28
MSE (${k}_{1}$)	20.21	39.22	77.92	390.97
MSE (${k}_{2}$)	15.09	31.69	65.57	346.29
MSE (${k}_{3}$)	3.79	3.72	3.69	3.82
MSE (${k}_{4}$)	3.22	3.28	**3.25**	3.45
MSE (${k}_{5}$)	3.28	3.30	3.28	3.44
MSE (${k}_{6}$)	**3.17**	3.34	3.30	**3.24**
MSE (${k}_{7}$)	3.31	3.60	3.61	3.35
MSE (${k}_{8}$)	3.18	3.32	3.28	3.27
MSE (${k}_{9}$)	4.11	4.00	4.00	4.00
MSE (${k}_{10}$)	22.40	50.46	104.45	548.54
MSE (${k}_{11}$)	4.00	4.00	4.00	4.00
MSE (${k}_{12}$)	3.77	3.75	3.74	3.78
MSE (${k}_{13}$)	20.61	42.82	85.51	441.10
MSE (${k}_{14}$)	23.84	48.57	96.04	488.17
MSE (${k}_{15}$)	3.24	**3.14**	3.34	10.66
MSE (${k}_{16}$)	3.81	3.86	3.91	3.97

**Table 2:** Estimated MSEs for $\gamma =2$, *p* = 3 and $n=50$. Bold value indicates minimum MSE.

$\rho$	0.8	0.9	0.95	0.99
MSE (MLE)	20.08	34.21	64.44	300.33
MSE (${k}_{1}$)	14.99	25.25	46.53	221.37
MSE (${k}_{2}$)	12.25	20.48	39.22	191.61
MSE (${k}_{3}$)	3.81	3.76	3.72	3.62
MSE (${k}_{4}$)	3.79	3.73	3.75	**3.42**
MSE (${k}_{5}$)	3.89	3.77	3.76	3.45
MSE (${k}_{6}$)	3.83	3.81	3.92	3.48
MSE (${k}_{7}$)	3.85	3.82	3.93	3.49
MSE (${k}_{8}$)	3.91	3.92	4.02	3.53
MSE (${k}_{9}$)	4.16	4.00	4.00	4.00
MSE (${k}_{10}$)	15.22	28.91	58.64	293.80
MSE (${k}_{11}$)	4.00	4.00	4.00	4.00
MSE (${k}_{12}$)	3.83	3.81	3.80	3.81
MSE (${k}_{13}$)	15.81	26.94	51.50	244.28
MSE (${k}_{14}$)	18.33	30.88	58.04	270.35
MSE (${k}_{15}$)	**3.65**	**3.46**	**3.37**	5.00
MSE (${k}_{16}$)	3.80	3.80	3.85	3.94

**Table 3:** Estimated MSEs for $\gamma =2$, *p* = 3 and $n=100$. Bold value indicates minimum MSE.

$\rho$	0.8	0.9	0.95	0.99
MSE (MLE)	20.00	31.21	51.69	236.58
MSE (${k}_{1}$)	15.44	23.36	37.24	170.50
MSE (${k}_{2}$)	13.22	19.64	32.26	151.58
MSE (${k}_{3}$)	3.90	3.87	**3.85**	**3.78**
MSE (${k}_{4}$)	5.67	5.56	5.38	4.44
MSE (${k}_{5}$)	5.81	5.61	5.28	4.18
MSE (${k}_{6}$)	5.71	5.64	5.57	4.87
MSE (${k}_{7}$)	5.75	5.64	5.56	4.88
MSE (${k}_{8}$)	5.74	5.69	5.62	4.86
MSE (${k}_{9}$)	4.70	4.06	4.00	4.00
MSE (${k}_{10}$)	15.24	26.36	47.03	231.93
MSE (${k}_{11}$)	4.00	4.00	4.00	4.00
MSE (${k}_{12}$)	**3.88**	**3.87**	3.86	3.84
MSE (${k}_{13}$)	16.53	25.45	42.13	195.54
MSE (${k}_{14}$)	18.59	28.62	46.98	214.09
MSE (${k}_{15}$)	4.32	4.22	4.11	4.01
MSE (${k}_{16}$)	4.14	3.95	3.86	3.91

**Table 4:** Estimated MSEs for $\gamma =2$, *p* = 3 and $n=150$. Bold value indicates minimum MSE.

$\rho$	0.8	0.9	0.95	0.99
MSE (MLE)	21.03	31.39	54.06	228.55
MSE (${k}_{1}$)	16.11	23.61	39.76	164.82
MSE (${k}_{2}$)	14.71	20.72	34.63	147.85
MSE (${k}_{3}$)	3.94	3.92	3.90	**3.84**
MSE (${k}_{4}$)	7.26	7.12	7.11	5.78
MSE (${k}_{5}$)	7.39	7.14	6.96	5.23
MSE (${k}_{6}$)	7.31	7.23	7.35	6.41
MSE (${k}_{7}$)	7.34	7.21	7.28	6.30
MSE (${k}_{8}$)	7.35	7.30	7.44	6.45
MSE (${k}_{9}$)	5.51	4.10	4.00	4.00
MSE (${k}_{10}$)	16.30	26.76	49.24	223.86
MSE (${k}_{11}$)	4.00	4.00	4.00	4.00
MSE (${k}_{12}$)	**3.89**	**3.89**	**3.88**	3.87
MSE (${k}_{13}$)	17.79	26.07	44.52	188.82
MSE (${k}_{14}$)	19.76	29.09	49.53	207.44
MSE (${k}_{15}$)	4.74	4.67	4.63	4.48
MSE (${k}_{16}$)	4.70	4.26	3.99	3.89

**Table 5:** Estimated MSEs for $\gamma =2$, *p* = 3 and $n=200$. Bold value indicates minimum MSE.

$\rho$	0.8	0.9	0.95	0.99
MSE (MLE)	23.24	31.77	52.15	222.61
MSE (${k}_{1}$)	18.30	24.21	38.84	162.73
MSE (${k}_{2}$)	17.26	21.99	34.45	144.35
MSE (${k}_{3}$)	3.96	3.95	3.94	3.90
MSE (${k}_{4}$)	9.50	9.21	9.10	7.63
MSE (${k}_{5}$)	9.62	9.23	8.91	6.76
MSE (${k}_{6}$)	9.55	9.32	9.34	8.44
MSE (${k}_{7}$)	9.60	9.30	9.28	8.34
MSE (${k}_{8}$)	9.57	9.36	9.42	8.44
MSE (${k}_{9}$)	6.78	4.37	4.01	4.00
MSE (${k}_{10}$)	18.48	27.11	47.50	217.78
MSE (${k}_{11}$)	4.00	4.00	4.00	4.00
MSE (${k}_{12}$)	**3.91**	**3.90**	**3.90**	**3.89**
MSE (${k}_{13}$)	20.30	26.85	43.46	185.60
MSE (${k}_{14}$)	22.08	29.70	48.12	202.84
MSE (${k}_{15}$)	5.19	5.13	5.12	5.04
MSE (${k}_{16}$)	5.59	4.87	4.30	3.89

**Table 6:** Estimated MSEs for $\gamma =4$, *p* = 3 and $n=25$. Bold value indicates minimum MSE.

$\rho$	0.8	0.9	0.95	0.99
MSE (MLE)	53.50	114.96	217.19	1133.97
MSE (${k}_{1}$)	39.72	84.54	152.68	824.22
MSE (${k}_{2}$)	28.95	67.72	129.65	705.63
MSE (${k}_{3}$)	3.85	3.80	3.78	4.00
MSE (${k}_{4}$)	**3.36**	**3.33**	3.38	3.61
MSE (${k}_{5}$)	3.46	3.43	3.49	3.69
MSE (${k}_{6}$)	3.37	3.35	3.35	**3.42**
MSE (${k}_{7}$)	3.57	3.65	3.66	3.44
MSE (${k}_{8}$)	**3.36**	**3.33**	**3.35**	3.48
MSE (${k}_{9}$)	4.00	4.00	4.00	4.00
MSE (${k}_{10}$)	45.75	106.95	209.00	1125.26
MSE (${k}_{11}$)	4.00	4.00	4.00	4.00
MSE (${k}_{12}$)	3.78	3.77	3.77	3.83
MSE (${k}_{13}$)	44.79	98.49	184.54	971.71
MSE (${k}_{14}$)	49.17	106.44	199.50	1042.32
MSE (${k}_{15}$)	3.37	3.97	4.73	23.21
MSE (${k}_{16}$)	3.89	3.93	3.95	3.98

**Table 7:** Estimated MSEs for $\gamma =4$, *p* = 3 and $n=50$. Bold value indicates minimum MSE.

$\rho$	0.8	0.9	0.95	0.99
MSE (MLE)	35.39	60.02	117.00	585.55
MSE (${k}_{1}$)	25.86	43.56	83.77	428.67
MSE (${k}_{2}$)	20.26	35.27	71.21	374.07
MSE (${k}_{3}$)	3.85	3.80	3.75	3.69
MSE (${k}_{4}$)	3.58	3.44	**3.39**	3.45
MSE (${k}_{5}$)	3.72	3.52	3.47	3.57
MSE (${k}_{6}$)	3.55	3.43	**3.39**	**3.33**
MSE (${k}_{7}$)	3.62	3.48	3.44	**3.33**
MSE (${k}_{8}$)	3.57	3.45	3.41	3.37
MSE (${k}_{9}$)	4.00	4.00	4.00	4.00
MSE (${k}_{10}$)	27.06	51.29	107.12	573.62
MSE (${k}_{11}$)	4.00	4.00	4.00	4.00
MSE (${k}_{12}$)	3.83	3.81	3.80	3.84
MSE (${k}_{13}$)	30.60	52.10	102.28	516.03
MSE (${k}_{14}$)	33.33	56.33	109.82	550.13
MSE (${k}_{15}$)	**3.48**	**3.30**	3.42	9.81
MSE (${k}_{16}$)	3.85	3.88	3.92	3.97

**Table 8:** Estimated MSEs for $\gamma =4$, *p* = 3 and $n=100$. Bold value indicates minimum MSE.

$\rho$	0.8	0.9	0.95	0.99
MSE (MLE)	32.53	54.07	98.74	471.14
MSE (${k}_{1}$)	23.48	38.42	70.71	338.15
MSE (${k}_{2}$)	18.50	31.74	59.93	301.75
MSE (${k}_{3}$)	3.88	3.85	3.83	3.74
MSE (${k}_{4}$)	4.14	4.22	4.02	**3.62**
MSE (${k}_{5}$)	4.31	4.23	3.93	3.67
MSE (${k}_{6}$)	4.06	4.19	4.03	3.66
MSE (${k}_{7}$)	4.09	4.22	4.07	3.71
MSE (${k}_{8}$)	4.08	4.19	4.02	3.64
MSE (${k}_{9}$)	4.00	4.00	4.00	4.00
MSE (${k}_{10}$)	24.07	45.64	90.12	461.99
MSE (${k}_{11}$)	4.00	4.00	4.00	4.00
MSE (${k}_{12}$)	3.87	3.85	3.84	3.84
MSE (${k}_{13}$)	28.47	47.70	87.72	421.95
MSE (${k}_{14}$)	30.78	51.11	93.26	445.32
MSE (${k}_{15}$)	3.88	**3.75**	**3.59**	5.08
MSE (${k}_{16}$)	**3.86**	3.86	3.89	3.96

**Table 9:** Estimated MSEs for $\gamma =4$, *p* = 3 and $n=150$. Bold value indicates minimum MSE.

$\rho$	0.8	0.9	0.95	0.99
MSE (MLE)	32.38	53.63	97.83	466.30
MSE (${k}_{1}$)	23.09	37.75	68.41	335.88
MSE (${k}_{2}$)	18.90	31.89	59.82	298.55
MSE (${k}_{3}$)	3.91	3.89	**3.85**	**3.78**
MSE (${k}_{4}$)	4.92	4.91	4.71	4.01
MSE (${k}_{5}$)	5.08	4.88	4.50	3.81
MSE (${k}_{6}$)	4.67`	4.88	4.75	4.18
MSE (${k}_{7}$)	4.89	4.91	4.77	4.24
MSE (${k}_{8}$)	4.88	4.90	4.73	4.11
MSE (${k}_{9}$)	4.00	4.00	4.00	4.00
MSE (${k}_{10}$)	23.90	45.06	88.96	456.50
MSE (${k}_{11}$)	4.00	4.00	4.00	4.00
MSE (${k}_{12}$)	**3.88**	**3.87**	3.86	3.86
MSE (${k}_{13}$)	28.62	47.64	87.16	417.31
MSE (${k}_{14}$)	30.78	50.88	92.64	441.85
MSE (${k}_{15}$)	4.21	4.09	3.91	4.51
MSE (${k}_{16}$)	3.93	3.88	3.88	3.95

**Table 10:** Estimated MSEs for $\gamma =4$, *p* = 3 and $n=200$. Bold value indicates minimum MSE.

$\rho$	0.8	0.9	0.95	0.99
MSE (MLE)	32.45	51.51	93.33	431.09
MSE (${k}_{1}$)	23.12	36.67	64.93	310.53
MSE (${k}_{2}$)	19.56	31.01	57.17	278.42
MSE (${k}_{3}$)	3.93	3.92	3.89	**3.83**
MSE (${k}_{4}$)	5.92	5.82	5.59	4.54
MSE (${k}_{5}$)	6.07	5.75	5.26	4.04
MSE (${k}_{6}$)	5.86	5.80	5.64	4.83
MSE (${k}_{7}$)	5.90	5.83	5.68	4.94
MSE (${k}_{8}$)	5.88	5.80	5.62	4.68
MSE (${k}_{9}$)	4.00	4.00	4.00	4.00
MSE (${k}_{10}$)	24.15	43.09	84.54	422.08
MSE (${k}_{11}$)	4.00	4.00	4.00	4.00
MSE (${k}_{12}$)	**3.89**	**3.88**	**3.88**	3.87
MSE (${k}_{13}$)	28.91	45.93	83.46	388.62
MSE (${k}_{14}$)	30.96	48.97	88.56	409.24
MSE (${k}_{15}$)	4.51	4.42	4.28	4.33
MSE (${k}_{16}$)	4.10	3.95	3.89	3.94

**Table 11:** Estimated MSEs for $\gamma =6$, *p* = 3 and $n=25$. Bold value indicates minimum MSE.

$\rho$	0.8	0.9	0.95	0.99
MSE (MLE)	391.26	170.79	35,737.33	1984.19
MSE (${k}_{1}$)	330.39	117.40	35,636.52	1511.99
MSE (${k}_{2}$)	296.87	98.87	35,563.03	1299.81
MSE (${k}_{3}$)	3.88	3.83	3.81	4.18
MSE (${k}_{4}$)	**3.49**	**3.42**	3.47	3.69
MSE (${k}_{5}$)	3.78	3.56	10.23	3.79
MSE (${k}_{6}$)	3.56	3.44	**3.40**	**3.54**
MSE (${k}_{7}$)	4.03	3.81	15.15	3.57
MSE (${k}_{8}$)	3.56	3.44	3.43	3.61
MSE (${k}_{9}$)	147.10	4.00	35,398.72	4.00
MSE (${k}_{10}$)	378.73	160.02	35,717.34	1971.73
MSE (${k}_{11}$)	4.00	4.00	4.00	4.00
MSE (${k}_{12}$)	3.80	3.78	3.79	3.85
MSE (${k}_{13}$)	236.25	149.79	302.05	1769.03
MSE (${k}_{14}$)	242.98	159.90	322.21	1863.72
MSE (${k}_{15}$)	12.53	4.97	8.25	44.18
MSE (${k}_{16}$)	3.93	3.95	3.97	3.99

**Table 12:** Estimated MSEs for $\gamma =6$, *p* = 3 and $n=50$. Bold value indicates minimum MSE.

$\rho$	0.8	0.9	0.95	0.99
MSE (MLE)	49.97	89.63	174.47	842.89
MSE (${k}_{1}$)	35.76	62.93	124.45	611.33
MSE (${k}_{2}$)	27.93	52.67	107.12	541.67
MSE (${k}_{3}$)	3.86	3.83	3.80	3.74
MSE (${k}_{4}$)	3.55	3.47	3.42	3.53
MSE (${k}_{5}$)	3.66	3.57	3.55	3.70
MSE (${k}_{6}$)	3.51	**3.45**	**3.40**	3.42
MSE (${k}_{7}$)	3.56	3.52	3.46	**3.38**
MSE (${k}_{8}$)	3.53	3.47	3.42	3.47
MSE (${k}_{9}$)	4.00	4.00	4.00	4.00
MSE (${k}_{10}$)	38.63	77.28	160.60	825.89
MSE (${k}_{11}$)	4.00	4.00	4.00	4.00
MSE (${k}_{12}$)	3.83	3.82	3.81	3.85
MSE (${k}_{13}$)	44.72	80.76	158.45	766.38
MSE (${k}_{14}$)	47.68	85.50	166.55	804.57
MSE (${k}_{15}$)	**3.45**	3.46	3.96	15.96
MSE (${k}_{16}$)	3.89	3.92	3.95	3.98

**Table 13:** Estimated MSEs for $\gamma =6$, *p* = 3 and $n=100$. Bold value indicates minimum MSE.

$\rho$	0.8	0.9	0.95	0.99
MSE (MLE)	45.49	76.39	145.71	690.49
MSE (${k}_{1}$)	31.77	53.94	104.51	497.79
MSE (${k}_{2}$)	25.05	44.48	88.55	445.01
MSE (${k}_{3}$)	3.89	3.86	3.84	3.77
MSE (${k}_{4}$)	4.03	3.87	3.79	3.57
MSE (${k}_{5}$)	4.10	3.89	3.76	3.73
MSE (${k}_{6}$)	3.96	3.81	3.77	**3.53**
MSE (${k}_{7}$)	3.97	3.82	3.81	**3.53**
MSE (${k}_{8}$)	3.99	3.83	3.77	3.56
MSE (${k}_{9}$)	4.00	4.00	4.00	4.00
MSE (${k}_{10}$)	33.49	64.39	133.25	677.32
MSE (${k}_{11}$)	4.00	4.00	4.00	4.00
MSE (${k}_{12}$)	3.87	3.85	3.84	3.84
MSE (${k}_{13}$)	41.29	69.81	133.76	636.66
MSE (${k}_{14}$)	43.63	73.27	139.80	662.86
MSE (${k}_{15}$)	**3.76**	**3.56**	**3.56**	7.44
MSE (${k}_{16}$)	3.88	3.89	3.93	3.98

**Table 14:** Estimated MSEs for $\gamma =6$, *p* = 3 and $n=150$. Bold value indicates minimum MSE.

$\rho$	0.8	0.9	0.95	0.99
MSE (MLE)	45.63	75.75	142.13	683.71
MSE (${k}_{1}$)	31.72	51.97	98.42	488.66
MSE (${k}_{2}$)	25.56	43.79	86.61	440.52
MSE (${k}_{3}$)	3.90	3.88	3.85	3.77
MSE (${k}_{4}$)	4.37	4.26	4.14	**3.70**
MSE (${k}_{5}$)	4.47	4.19	3.97	3.74
MSE (${k}_{6}$)	4.28	4.20	4.13	3.73
MSE (${k}_{7}$)	4.26	4.20	4.17	3.78
MSE (${k}_{8}$)	4.33	4.23	4.12	3.71
MSE (${k}_{9}$)	4.00	4.00	4.00	4.00
MSE (${k}_{10}$)	33.94	63.69	129.32	669.99
MSE (${k}_{11}$)	4.00	4.00	4.00	4.00
MSE (${k}_{12}$)	**3.87**	3.87	3.86	3.86
MSE (${k}_{13}$)	41.72	69.43	130.86	632.26
MSE (${k}_{14}$)	43.91	72.80	136.71	658.14
MSE (${k}_{15}$)	3.96	**3.80**	**3.73**	6.23
MSE (${k}_{16}$)	3.88	3.88	3.91	3.97

**Table 15:** Estimated MSEs for $\gamma =6$, *p* = 3 and $n=200$. Bold value indicates minimum MSE.

$\rho$	0.8	0.9	0.95	0.99
MSE (MLE)	45.16	74.59	132.27	629.67
MSE (${k}_{1}$)	30.87	50.58	92.99	450.02
MSE (${k}_{2}$)	25.46	43.55	80.39	404.32
MSE (${k}_{3}$)	3.91	3.90	**3.88**	**3.81**
MSE (${k}_{4}$)	5.02	4.83	4.72	3.95
MSE (${k}_{5}$)	5.05	4.66	4.35	3.79
MSE (${k}_{6}$)	4.94	4.78	4.73	4.06
MSE (${k}_{7}$)	4.94	4.78	4.77	4.18
MSE (${k}_{8}$)	4.99	4.80	4.70	3.96
MSE (${k}_{9}$)	4.00	4.00	4.00	4.00
MSE (${k}_{10}$)	33.03	62.37	120.04	616.36
MSE (${k}_{11}$)	4.00	4.00	4.00	4.00
MSE (${k}_{12}$)	**3.89**	**3.88**	3.87	3.86
MSE (${k}_{13}$)	41.50	68.70	122.18	583.40
MSE (${k}_{14}$)	43.53	71.83	127.32	606.42
MSE (${k}_{15}$)	4.23	4.09	3.98	5.09
MSE (${k}_{16}$)	3.92	**3.88**	3.90	3.96

**Table 16:** Estimated MSEs for $\gamma =2$, *p* = 6 and $n=25$. Bold value indicates minimum MSE.

$\rho$	0.8	0.9	0.95	0.99
MSE (MLE)	76.14	150.55	299.74	1618.96
MSE (${k}_{1}$)	58.88	115.88	231.17	1221.04
MSE (${k}_{2}$)	44.03	88.93	183.05	1003.77
MSE (${k}_{3}$)	3.77	3.71	3.66	4.46
MSE (${k}_{4}$)	**2.96**	**3.03**	**3.00**	**3.22**
MSE (${k}_{5}$)	3.01	3.12	3.16	3.28
MSE (${k}_{6}$)	4.38	4.91	4.85	3.67
MSE (${k}_{7}$)	5.54	6.72	6.90	4.90
MSE (${k}_{8}$)	5.87	6.72	6.80	4.73
MSE (${k}_{9}$)	4.05	4.02	4.02	4.02
MSE (${k}_{10}$)	68.03	141.44	290.08	1608.90
MSE (${k}_{11}$)	4.02	4.02	4.02	4.02
MSE (${k}_{12}$)	3.65	3.61	3.61	3.70
MSE (${k}_{13}$)	59.99	119.01	239.52	1304.66
MSE (${k}_{14}$)	70.31	138.27	275.68	1488.70
MSE (${k}_{15}$)	**2.96**	4.42	9.94	77.98
MSE (${k}_{16}$)	3.90	3.94	3.96	4.00

**Table 17:** Estimated MSEs for $\gamma =2$, *p* = 6 and $n=50$. Bold value indicates minimum MSE.

$\rho$	0.8	0.9	0.95	0.99
MSE (MLE)	46.76	81.80	153.27	755.87
MSE (${k}_{1}$)	36.79	63.53	121.70	592.38
MSE (${k}_{2}$)	28.59	51.48	99.19	503.96
MSE (${k}_{3}$)	3.80	3.71	3.65	3.78
MSE (${k}_{4}$)	3.46	3.72	3.61	**3.07**
MSE (${k}_{5}$)	3.15	3.22	**3.15**	**3.07**
MSE (${k}_{6}$)	4.63	5.47	5.66	4.11
MSE (${k}_{7}$)	5.23	6.37	6.80	4.79
MSE (${k}_{8}$)	6.92	8.58	9.61	6.95
MSE (${k}_{9}$)	4.02	4.02	4.02	4.02
MSE (${k}_{10}$)	39.43	74.81	146.27	748.74
MSE (${k}_{11}$)	4.02	4.02	4.02	4.02
MSE (${k}_{12}$)	3.73	3.68	3.65	3.66
MSE (${k}_{13}$)	38.31	68.04	129.22	645.19
MSE (${k}_{14}$)	44.25	77.30	144.94	715.24
MSE (${k}_{15}$)	**2.97**	**2.94**	4.09	26.90
MSE (${k}_{16}$)	3.84	3.88	3.92	3.98

**Table 18:** Estimated MSEs for $\gamma =2$, *p* = 6 and $n=100$. Bold value indicates minimum MSE.

$\rho$	0.8	0.9	0.95	0.99
MSE (MLE)	42.16	71.11	130.55	630.34
MSE (${k}_{1}$)	33.99	56.54	102.90	499.44
MSE (${k}_{2}$)	26.94	45.70	85.22	422.87
MSE (${k}_{3}$)	3.87	3.82	3.77	3.71
MSE (${k}_{4}$)	5.51	5.61	5.59	3.86
MSE (${k}_{5}$)	4.91	4.61	4.36	**3.37**
MSE (${k}_{6}$)	6.39	6.96	7.31	4.90
MSE (${k}_{7}$)	6.65	7.20	7.58	5.04
MSE (${k}_{8}$)	8.72	10.28	11.71	8.57
MSE (${k}_{9}$)	4.02	4.02	4.02	4.02
MSE (${k}_{10}$)	35.30	64.52	124.20	623.99
MSE (${k}_{11}$)	4.02	4.02	4.02	4.02
MSE (${k}_{12}$)	**3.83**	3.80	3.77	3.74
MSE (${k}_{13}$)	35.53	60.26	112.00	545.43
MSE (${k}_{14}$)	40.25	67.72	124.21	599.64
MSE (${k}_{15}$)	3.88	**3.62**	**3.65**	10.14
MSE (${k}_{16}$)	**3.83**	3.85	3.89	3.97

The simulation findings extracted from Tables 1, 2, 3, 4, 5, 6, 7, 8, 9, 10, 11, 12, 13, 14, 15, 16, 17, 18, 19, 20, 21, 22, 23, 24, 25, 26, 27, 28, 29, 30, 31, 32, 33, 34, 35, 36 are summarized as follows:

(i) The basic purpose of this simulation study is to study the performance of our proposed RPEs for the QPRRE in the presence of multicollinearity. As the multicollinearity level increases with fixed the number of predictors, the sample size, and dispersion, the estimated MSEs increase for all the estimation methods under study. However, at the high level of multicollinearity and with a larger sample size, mostly the QPRRE with RPEs ${\widehat{k}}_{3},\, {\widehat{k}}_{5},\, {\widehat{k}}_{9},\, {\widehat{k}}_{11},\, {\widehat{k}}_{12}\, and \,{\widehat{k}}_{16}$ gives the smaller MSEs as compared to the QPRRE with other RPEs and the QLE.
(ii) The other factor that may affect the estimated MSEs of the QPRRE and the QLE is the number of regressors. According to the results of the simulation, we noticed that the estimated MSEs of the estimators and the number of regressors are directly proportional; it means that when the regressors increase by fixing the other factors, the estimated MSEs of QPRRE and the QLE also increase.
(iii) When we see the effect of sample size on the MSEs of the estimators, it is observed that the relation between the estimated MSEs and the sample size is inverse. As the sample size increases by fixing the other factors the estimated MSEs decrease. Results show that mostly the QPRRE with RPEs ${\widehat{k}}_{3},\, {\widehat{k}}_{5},\, {\widehat{k}}_{9},\, {\widehat{k}}_{11},\, {\widehat{k}}_{12} \,and\, {\widehat{k}}_{16}$ performs well as compared to the other RPEs and the QLE based on minimum estimated MSE.
(iv) According to the results, in every situation such as the different combinations of multicollinearity, different sample size, different number of explanatory variables and different levels of dispersion, the proposed QPRRE with different RPEs outperforms the QLE based on the minimum MSE.
(v) According to the findings of simulation for all the conditions under study, the estimated MSE of the QLE is always greater than all suggested RPEs for QPRRE. We also noticed that the suggested QPRRE is significantly decreasing the estimated MSE. Finally, it is concluded that the proposed RPEs for the QPRRE perform well and give better results than the QLE due to the minimum estimated MSE under certain conditions. Mostly, the QPRRE with RPEs ${\widehat{k}}_{3},\, {\widehat{k}}_{5},\, {\widehat{k}}_{9},\, {\widehat{k}}_{11},\, {\widehat{k}}_{12}\, and\, {\widehat{k}}_{16}$ gives better results than the QPRRE with other RPEs. There are some situations, where the QPRRE with other RPEs i.e. ${\widehat{k}}_{4}{-}{\widehat{k}}_{8},\, and\, {\widehat{k}}_{15}$ gives better performance than others. By the evidence of simulation results, the study that is reported in this work is that the QPRR outperforms the QLE in the presence of multicollinearity. So, we suggest the researchers use QPRRE with the best biasing parameters ${\widehat{k}}_{3},\, {\widehat{k}}_{5},\,{\widehat{k}}_{9},\, {\widehat{k}}_{11},\, {\widehat{k}}_{12} \,and\, {\widehat{k}}_{16}$ that minimize the effects of the multicollinearity in QPRM due to their robust patterns.

**Table 19:** Estimated MSEs for $\gamma =2$, *p* = 6 and $n=150$. Bold value indicates minimum MSE.

$\rho$	0.8	0.9	0.95	0.99
MSE (MLE)	43.41	70.93	128.81	600.58
MSE (${k}_{1}$)	34.62	56.58	101.89	475.26
MSE (${k}_{2}$)	28.92	46.94	85.64	406.93
MSE (${k}_{3}$)	3.91	3.87	**3.82**	**3.74**
MSE (${k}_{4}$)	7.50	7.80	7.58	5.14
MSE (${k}_{5}$)	6.66	6.44	5.92	4.01
MSE (${k}_{6}$)	8.39	9.23	9.47	6.52
MSE (${k}_{7}$)	8.71	9.53	9.83	6.76
MSE (${k}_{8}$)	10.83	12.84	14.47	10.88
MSE (${k}_{9}$)	4.02	4.02	4.02	4.02
MSE (${k}_{10}$)	36.46	64.30	122.39	594.25
MSE (${k}_{11}$)	4.02	4.02	4.02	4.02
MSE (${k}_{12}$)	**3.87**	**3.84**	3.83	3.79
MSE (${k}_{13}$)	37.16	60.75	111.16	522.54
MSE (${k}_{14}$)	41.66	67.83	123.00	572.85
MSE (${k}_{15}$)	4.45	4.29	4.24	6.84
MSE (${k}_{16}$)	3.88	**3.84**	3.87	3.96

**Table 20:** Estimated MSEs for $\gamma =2$, *p* = 6 and $n=200$. Bold value indicates minimum MSE.

$\rho$	0.8	0.9	0.95	0.99
MSE (MLE)	45.60	71.45	126.89	581.02
MSE (${k}_{1}$)	36.81	56.72	99.72	461.11
MSE (${k}_{2}$)	31.70	48.31	85.17	393.02
MSE (${k}_{3}$)	3.94	3.92	3.90	**3.82**
MSE (${k}_{4}$)	10.02	9.94	9.83	6.79
MSE (${k}_{5}$)	9.15	8.47	7.91	5.02
MSE (${k}_{6}$)	10.84	11.25	11.57	8.17
MSE (${k}_{7}$)	11.26	11.63	11.95	8.38
MSE (${k}_{8}$)	13.16	14.88	16.77	13.01
MSE (${k}_{9}$)	4.02	4.02	4.02	4.02
MSE (${k}_{10}$)	38.57	64.76	120.22	574.52
MSE (${k}_{11}$)	4.02	4.02	4.02	4.02
MSE (${k}_{12}$)	**3.90**	**3.88**	3.87	3.83
MSE (${k}_{13}$)	39.69	61.59	109.64	505.43
MSE (${k}_{14}$)	43.98	68.54	121.37	554.59
MSE (${k}_{15}$)	5.09	4.95	4.90	5.50
MSE (${k}_{16}$)	4.05	3.89	**3.86**	3.95

**Table 21:** Estimated MSEs for $\gamma =4$, *p* = 6 and $n=25$. Bold value indicates minimum MSE.

$\rho$	0.8	0.9	0.95	0.99
MSE (MLE)	2029.91	352.30	726.27	9290.28
MSE (${k}_{1}$)	1975.04	261.89	545.28	8376.94
MSE (${k}_{2}$)	1937.11	204.38	431.91	7875.11
MSE (${k}_{3}$)	3.84	3.80	3.79	4.72
MSE (${k}_{4}$)	**3.22**	**3.15**	**3.21**	**3.45**
MSE (${k}_{5}$)	12.29	3.21	3.35	9.76
MSE (${k}_{6}$)	4.76	4.37	4.16	3.57
MSE (${k}_{7}$)	15.38	6.97	6.81	11.04
MSE (${k}_{8}$)	5.53	5.23	4.89	3.88
MSE (${k}_{9}$)	1818.23	4.02	4.02	5584.79
MSE (${k}_{10}$)	2014.17	336.55	709.56	9272.98
MSE (${k}_{11}$)	4.02	4.02	4.02	4.02
MSE (${k}_{12}$)	3.69	3.66	3.68	3.79
MSE (${k}_{13}$)	190.12	293.66	607.23	3149.96
MSE (${k}_{14}$)	209.85	329.17	675.86	3488.69
MSE (${k}_{15}$)	6.75	11.02	26.47	183.91
MSE (${k}_{16}$)	3.96	3.97	3.99	4.00

**Table 22:** Estimated MSEs for $\gamma =4$, *p* = 6 and $n=50$. Bold value indicates minimum MSE.

$\rho$	0.8	0.9	0.95	0.99
MSE (MLE)	85.06	152.04	301.30	1481.69
MSE (${k}_{1}$)	66.84	119.97	232.90	1145.13
MSE (${k}_{2}$)	51.27	95.37	193.43	979.51
MSE (${k}_{3}$)	3.85	3.79	3.74	3.87
MSE (${k}_{4}$)	3.17	3.25	3.25	**3.17**
MSE (${k}_{5}$)	3.06	**3.03**	**3.12**	3.40
MSE (${k}_{6}$)	3.90	4.25	4.19	3.47
MSE (${k}_{7}$)	4.88	5.62	5.58	4.18
MSE (${k}_{8}$)	5.19	5.80	5.71	4.27
MSE (${k}_{9}$)	4.02	4.02	4.02	4.02
MSE (${k}_{10}$)	72.97	139.71	288.48	1468.52
MSE (${k}_{11}$)	4.02	4.02	4.02	4.02
MSE (${k}_{12}$)	3.74	3.69	3.67	3.71
MSE (${k}_{13}$)	75.50	136.12	270.95	1342.56
MSE (${k}_{14}$)	82.09	146.83	291.06	1433.17
MSE (${k}_{15}$)	**3.00**	3.79	7.86	58.93
MSE (${k}_{16}$)	3.93	3.95	3.97	4.00

**Table 23:** Estimated MSEs for $\gamma =4$, *p* = 6 and $n=100$. Bold value indicates minimum MSE.

$\rho$	0.8	0.9	0.95	0.99
MSE (MLE)	73.20	132.25	245.28	1231.12
MSE (${k}_{1}$)	57.22	104.53	191.97	964.83
MSE (${k}_{2}$)	44.64	83.46	159.28	826.35
MSE (${k}_{3}$)	3.88	3.83	3.77	3.76
MSE (${k}_{4}$)	3.73	3.85	3.67	**3.19**
MSE (${k}_{5}$)	3.57	3.45	**3.31**	3.41
MSE (${k}_{6}$)	4.24	4.57	4.38	3.40
MSE (${k}_{7}$)	4.93	5.49	5.29	3.78
MSE (${k}_{8}$)	5.47	6.07	6.01	4.03
MSE (${k}_{9}$)	4.02	4.02	4.02	4.02
MSE (${k}_{10}$)	61.78	120.65	233.86	1219.09
MSE (${k}_{11}$)	4.02	4.02	4.02	4.02
MSE (${k}_{12}$)	3.81	3.77	3.74	3.74
MSE (${k}_{13}$)	66.15	120.44	224.07	1131.91
MSE (${k}_{14}$)	71.07	128.44	238.24	1197.07
MSE (${k}_{15}$)	**3.36**	**3.31**	4.39	30.46
MSE (${k}_{16}$)	3.90	3.93	3.96	3.99

**Table 24:** Estimated MSEs for $\gamma =4$, *p* = 6 and $n=150$. Bold value indicates minimum MSE.

$\rho$	0.8	0.9	0.95	0.99
MSE (MLE)	74.48	126.81	240.14	1175.71
MSE (${k}_{1}$)	58.19	99.27	184.92	931.55
MSE (${k}_{2}$)	46.35	81.39	157.25	793.87
MSE (${k}_{3}$)	3.89	3.84	3.80	3.73
MSE (${k}_{4}$)	4.73	4.67	4.34	3.44
MSE (${k}_{5}$)	4.33	3.96	**3.65**	**3.42**
MSE (${k}_{6}$)	5.28	5.47	5.13	3.76
MSE (${k}_{7}$)	6.17	6.55	6.28	4.31
MSE (${k}_{8}$)	6.71	7.24	6.93	4.51
MSE (${k}_{9}$)	4.02	4.02	4.02	4.02
MSE (${k}_{10}$)	62.54	115.12	228.19	1163.69
MSE (${k}_{11}$)	4.02	4.02	4.02	4.02
MSE (${k}_{12}$)	3.84	3.81	3.79	3.77
MSE (${k}_{13}$)	67.79	116.02	220.34	1086.53
MSE (${k}_{14}$)	72.52	123.43	233.74	1145.40
MSE (${k}_{15}$)	**3.75**	**3.58**	4.15	20.20
MSE (${k}_{16}$)	3.88	3.91	3.94	3.99

**Table 25:** Estimated MSEs for $\gamma =4$, *p* = 6 and $n=200$. Bold value indicates minimum MSE.

$\rho$	0.8	0.9	0.95	0.99
MSE (MLE)	73.61	125.90	235.38	1125.75
MSE (${k}_{1}$)	56.61	98.17	181.70	886.62
MSE (${k}_{2}$)	46.07	81.18	154.47	761.00
MSE (${k}_{3}$)	3.91	3.87	3.83	3.77
MSE (${k}_{4}$)	5.48	5.53	5.25	3.84
MSE (${k}_{5}$)	5.07	4.70	4.30	**3.53**
MSE (${k}_{6}$)	5.98	6.25	5.98	4.18
MSE (${k}_{7}$)	6.85	7.36	7.15	4.73
MSE (${k}_{8}$)	7.46	8.17	7.80	5.01
MSE (${k}_{9}$)	4.02	4.02	4.02	4.02
MSE (${k}_{10}$)	61.53	113.67	223.26	1113.51
MSE (${k}_{11}$)	4.02	4.02	4.02	4.02
MSE (${k}_{12}$)	**3.86**	**3.84**	**3.82**	3.79
MSE (${k}_{13}$)	67.04	115.26	216.48	1041.61
MSE (${k}_{14}$)	71.73	122.69	229.35	1097.64
MSE (${k}_{15}$)	4.11	3.93	4.08	12.73
MSE (${k}_{16}$)	3.88	3.90	3.93	3.99

**Table 26:** Estimated MSEs for $\gamma =6$, *p* = 6 and $n=25$. Bold value indicates minimum MSE.

$\rho$	0.8	0.9	0.95	0.99
MSE (MLE)	10,699.31	5363.77	24,305.02	4,600,807.50
MSE (${k}_{1}$)	10,499.65	4777.43	23,548.71	4,586,701.24
MSE (${k}_{2}$)	10,371.97	4824.64	23,303.58	4,559,009.54
MSE (${k}_{3}$)	3.87	7.12	6.34	13.21
MSE (${k}_{4}$)	**3.59**	**3.36**	**3.38**	**3.57**
MSE (${k}_{5}$)	27.23	8.36	30.51	67.37
MSE (${k}_{6}$)	6.27	4.61	4.71	**3.57**
MSE (${k}_{7}$)	31.20	12.39	34.34	86.94
MSE (${k}_{8}$)	7.00	5.31	5.42	3.74
MSE (${k}_{9}$)	9498.04	686.75	9803.50	4,581,285.97
MSE (${k}_{10}$)	10,659.59	5335.16	24,280.68	4,588,957.33
MSE (${k}_{11}$)	4.02	7.31	6.16	4.02
MSE (${k}_{12}$)	3.72	3.71	3.73	3.82
MSE (${k}_{13}$)	1013.29	4561.93	14,310.77	6994.35
MSE (${k}_{14}$)	1118.43	4628.56	14,433.48	7774.15
MSE (${k}_{15}$)	35.27	206.39	455.05	405.12
MSE (${k}_{16}$)	3.98	3.99	4.00	4.01

**Table 27:** Estimated MSEs for $\gamma =6$, *p* = 6 and $n=50$. Bold value indicates minimum MSE.

$\rho$	0.8	0.9	0.95	0.99
MSE (MLE)	125.25	232.19	443.06	2135.45
MSE (${k}_{1}$)	97.12	177.90	340.47	1673.18
MSE (${k}_{2}$)	75.38	145.22	283.82	1407.87
MSE (${k}_{3}$)	3.88	3.82	3.78	3.97
MSE (${k}_{4}$)	3.25	3.29	**3.26**	**3.30**
MSE (${k}_{5}$)	**3.24**	**3.22**	3.32	3.59
MSE (${k}_{6}$)	3.71	3.90	3.81	3.43
MSE (${k}_{7}$)	4.79	5.36	5.24	4.12
MSE (${k}_{8}$)	4.43	4.70	4.53	3.79
MSE (${k}_{9}$)	4.02	4.02	4.02	4.02
MSE (${k}_{10}$)	108.73	214.98	425.45	2117.17
MSE (${k}_{11}$)	4.02	4.02	4.02	4.02
MSE (${k}_{12}$)	3.75	3.71	3.70	3.75
MSE (${k}_{13}$)	114.19	213.31	407.97	1974.49
MSE (${k}_{14}$)	121.76	225.97	431.26	2080.25
MSE (${k}_{15}$)	3.35	5.21	11.57	91.98
MSE (${k}_{16}$)	3.96	3.97	3.99	4.00

**Table 28:** Estimated MSEs for $\gamma =6$, *p* = 6 and $n=100$. Bold value indicates minimum MSE.

$\rho$	0.8	0.9	0.95	0.99
MSE (MLE)	106.47	189.51	360.37	1785.46
MSE (${k}_{1}$)	82.47	146.48	280.76	1415.35
MSE (${k}_{2}$)	64.36	119.93	234.78	1199.01
MSE (${k}_{3}$)	3.90	3.85	3.80	3.78
MSE (${k}_{4}$)	3.45	3.44	**3.32**	**3.21**
MSE (${k}_{5}$)	3.48	**3.36**	3.35	3.58
MSE (${k}_{6}$)	3.72	3.81	3.65	3.26
MSE (${k}_{7}$)	4.57	4.87	4.66	3.63
MSE (${k}_{8}$)	4.32	4.51	4.24	3.48
MSE (${k}_{9}$)	4.02	4.02	4.02	4.02
MSE (${k}_{10}$)	90.25	173.51	344.15	1768.58
MSE (${k}_{11}$)	4.02	4.02	4.02	4.02
MSE (${k}_{12}$)	3.81	3.77	3.75	3.75
MSE (${k}_{13}$)	98.84	176.98	337.78	1678.78
MSE (${k}_{14}$)	104.14	185.49	352.85	1749.46
MSE (${k}_{15}$)	**3.28**	3.62	6.25	49.24
MSE (${k}_{16}$)	3.94	3.96	3.98	4.00

**Table 29:** Estimated MSEs for $\gamma =6$, *p* = 6 and $n=150$. Bold value indicates minimum MSE.

$\rho$	0.8	0.9	0.95	0.99
MSE (MLE)	105.17	186.90	354.16	1739.52
MSE (${k}_{1}$)	80.47	147.63	274.28	1360.92
MSE (${k}_{2}$)	64.58	119.62	232.60	1172.24
MSE (${k}_{3}$)	3.90	3.84	3.81	3.74
MSE (${k}_{4}$)	3.88	3.74	3.61	**3.24**
MSE (${k}_{5}$)	3.80	**3.52**	**3.44**	3.56
MSE (${k}_{6}$)	4.18	4.13	3.96	3.31
MSE (${k}_{7}$)	5.22	5.42	5.19	3.77
MSE (${k}_{8}$)	4.90	4.95	4.62	3.50
MSE (${k}_{9}$)	4.02	4.02	4.02	4.02
MSE (${k}_{10}$)	88.61	169.93	337.05	1721.84
MSE (${k}_{11}$)	4.02	4.02	4.02	4.02
MSE (${k}_{12}$)	3.84	3.80	3.78	3.77
MSE (${k}_{13}$)	98.21	175.37	333.40	1643.82
MSE (${k}_{14}$)	103.09	183.30	347.46	1707.36
MSE (${k}_{15}$)	**3.49**	3.62	5.49	39.70
MSE (${k}_{16}$)	3.93	3.95	3.97	4.00

**Table 30:** Estimated MSEs for $\gamma =6$, *p* = 6 and $n=200$. Bold value indicates minimum MSE.

$\rho$	0.8	0.9	0.95	0.99
MSE (MLE)	105.20	183.78	343.91	1659.09
MSE (${k}_{1}$)	81.01	141.70	268.41	1314.73
MSE (${k}_{2}$)	64.76	118.06	225.69	1122.87
MSE (${k}_{3}$)	3.90	3.87	3.83	3.75
MSE (${k}_{4}$)	4.33	4.18	4.08	**3.36**
MSE (${k}_{5}$)	4.21	3.86	**3.68**	3.57
MSE (${k}_{6}$)	4.57	4.52	4.39	3.44
MSE (${k}_{7}$)	5.60	5.78	5.62	3.86
MSE (${k}_{8}$)	5.27	5.27	5.01	3.62
MSE (${k}_{9}$)	4.02	4.02	4.02	4.02
MSE (${k}_{10}$)	87.87	167.19	326.49	1641.49
MSE (${k}_{11}$)	4.02	4.02	4.02	4.02
MSE (${k}_{12}$)	3.85	3.83	3.81	3.79
MSE (${k}_{13}$)	98.47	172.90	324.34	1570.36
MSE (${k}_{14}$)	103.23	180.40	337.70	1629.80
MSE (${k}_{15}$)	**3.74**	**3.67**	4.66	27.15
MSE (${k}_{16}$)	3.91	3.94	3.96	4.00

**Table 31:** Estimated MSEs for $\gamma =2$, $p=12$, and $n={50,\,100}$. Bold value indicates minimum MSE.

	*n* = 50				*n* = 100			
$\rho$	0.8	0.9	0.95	0.99	0.8	0.9	0.95	0.99
MSE (QLE)	166.47	324.93	640.92	3295.88	107.64	192.52	362.47	1802.61
MSE (${k}_{1}$)	135.63	262.20	520.15	2599.75	90.12	160.39	303.12	1493.34
MSE (${k}_{2}$)	102.47	203.67	409.34	2126.72	69.39	126.30	241.98	1221.72
MSE (${k}_{3}$)	3.77	3.69	3.77	5.56	3.83	3.76	3.71	4.23
MSE (${k}_{4}$)	8.12	9.38	8.79	4.84	9.72	11.77	11.91	6.78
MSE (${k}_{5}$)	6.37	7.26	6.92	4.19	5.82	6.21	5.96	3.87
MSE (${k}_{6}$)	19.28	25.95	28.31	17.41	15.97	22.08	25.68	16.89
MSE (${k}_{7}$)	24.79	35.61	41.91	29.53	18.29	26.20	32.06	23.20
MSE (${k}_{8}$)	34.95	53.63	71.00	65.31	28.69	43.83	63.79	72.98
MSE (${k}_{9}$)	4.01	4.01	4.01	4.01	4.01	4.01	4.01	4.01
MSE (${k}_{10}$)	154.33	312.70	629.08	3283.59	96.14	181.40	351.58	1791.69
MSE (${k}_{11}$)	4.01	4.01	4.01	4.01	4.01	4.01	4.01	4.01
MSE (${k}_{12}$)	**3.50**	**3.45**	**3.46**	**3.62**	3.69	**3.61**	**3.56**	**3.58**
MSE (${k}_{13}$)	138.87	273.23	542.37	2807.63	92.74	167.45	317.57	1588.01
MSE (${k}_{14}$)	160.07	312.25	616.23	3169.38	104.64	187.05	352.28	1752.20
MSE (${k}_{15}$)	5.30	17.77	53.31	405.38	**3.13**	5.11	13.78	129.13
MSE (${k}_{16}$)	3.93	3.95	3.97	3.99	3.91	3.93	3.95	3.99

**Table 32:** Estimated MSEs for $\gamma =2$, $p=12$ and $n={150,\, 200}$. Bold value indicates minimum MSE.

	*n* = 150				*n* = 200			
$\rho$	0.8	0.9	0.95	0.99	0.8	0.9	0.95	0.99
MSE (QLE)	107.05	189.61	357.70	1727.66	105.30	187.00	345.40	1667.68
MSE (${k}_{1}$)	90.21	158.82	300.00	1434.63	88.71	156.26	290.43	1389.27
MSE (${k}_{2}$)	70.74	126.52	240.97	1185.68	70.57	125.74	234.82	1148.19
MSE (${k}_{3}$)	3.85	3.81	3.73	3.87	3.89	3.83	3.79	3.78
MSE (${k}_{4}$)	11.89	14.37	14.29	7.91	13.63	15.86	15.90	8.43
MSE (${k}_{5}$)	6.73	7.03	6.20	3.77	8.10	7.87	7.07	4.07
MSE (${k}_{6}$)	16.94	22.47	24.90	15.31	18.09	23.06	25.56	15.18
MSE (${k}_{7}$)	18.89	25.91	29.99	19.57	19.97	26.53	31.02	19.86
MSE (${k}_{8}$)	30.40	45.54	64.67	72.84	31.71	46.16	65.99	72.88
MSE (${k}_{9}$)	4.01	4.01	4.01	4.01	4.01	4.01	4.01	4.01
MSE (${k}_{10}$)	95.86	178.75	347.13	1717.34	94.04	176.12	334.92	1657.31
MSE (${k}_{11}$)	4.01	4.01	4.01	4.01	4.01	4.01	4.01	4.01
MSE (${k}_{12}$)	3.77	**3.71**	**3.67**	**3.65**	**3.82**	**3.78**	**3.74**	**3.70**
MSE (${k}_{13}$)	93.40	166.66	316.09	1538.48	92.42	164.94	306.06	1487.39
MSE (${k}_{14}$)	104.42	184.81	348.54	1684.20	102.87	182.48	336.97	1626.90
MSE (${k}_{15}$)	**3.56**	4.21	8.25	75.10	4.13	4.16	5.93	39.80
MSE (${k}_{16}$)	3.90	3.92	3.95	3.99	3.88	3.91	3.94	3.99

**Table 33:** Estimated MSEs for $\gamma =4$, $p=12$, and $n={50,\,100}$. Bold value indicates minimum MSE.

	*n* = 50				*n* = 100			
$\rho$	0.8	0.9	0.95	0.99	0.8	0.9	0.95	0.99
MSE (QLE)	345.69	670.91	1385.28	6817.89	197.83	366.72	703.14	3463.65
MSE (${k}_{1}$)	276.99	534.72	1086.54	5336.42	164.59	302.52	574.74	2835.09
MSE (${k}_{2}$)	208.18	409.60	851.01	4281.11	126.07	238.77	465.21	2329.46
MSE (${k}_{3}$)	3.82	3.79	3.84	6.38	3.88	3.81	3.77	4.08
MSE (${k}_{4}$)	6.71	7.16	6.27	3.85	7.26	8.24	7.67	4.59
MSE (${k}_{5}$)	4.20	4.35	4.05	**3.44**	**3.47**	3.65	**3.55**	**3.29**
MSE (${k}_{6}$)	16.59	19.79	18.70	9.54	13.12	16.33	16.17	8.91
MSE (${k}_{7}$)	26.86	34.84	36.09	20.49	18.88	25.38	26.82	15.80
MSE (${k}_{8}$)	33.46	44.25	47.18	30.30	27.57	38.19	44.30	29.89
MSE (${k}_{9}$)	4.01	4.01	4.01	4.01	4.01	4.01	4.01	4.01
MSE (${k}_{10}$)	322.90	648.67	1362.17	6795.08	178.10	346.64	683.22	3443.42
MSE (${k}_{11}$)	4.01	4.01	4.01	4.01	4.01	4.01	4.01	4.01
MSE (${k}_{12}$)	**3.55**	**3.53**	**3.56**	3.71	3.68	**3.60**	3.57	3.64
MSE (${k}_{13}$)	305.08	592.96	1226.21	6081.90	180.95	336.98	647.91	3201.22
MSE (${k}_{14}$)	336.39	652.51	1346.84	6638.19	194.36	360.38	691.00	3405.41
MSE (${k}_{15}$)	13.14	39.76	116.06	801.41	3.95	11.06	32.74	295.64
MSE (${k}_{16}$)	3.97	3.98	3.99	4.00	3.96	3.97	3.98	4.00

**Table 34:** Estimated MSEs for $\gamma =4$, $p=12$, and $n={150,\,200}$. Bold value indicates minimum MSE.

	*n* = 150				*n* = 200			
$\rho$	0.8	0.9	0.95	0.99	0.8	0.9	0.95	0.99
MSE (QLE)	193.38	357.10	677.74	3351.75	191.55	348.17	659.99	3232.57
MSE (${k}_{1}$)	160.42	295.61	558.95	2796.27	160.42	287.85	551.46	2697.71
MSE (${k}_{2}$)	125.88	237.23	456.19	2282.88	125.67	232.71	447.39	2222.25
MSE (${k}_{3}$)	3.88	3.82	3.77	3.94	3.88	3.84	3.79	3.85
MSE (${k}_{4}$)	8.28	9.06	8.19	4.73	8.79	9.56	8.56	4.65
MSE (${k}_{5}$)	3.64	**3.54**	**3.36**	**3.23**	3.93	3.78	**3.51**	**3.23**
MSE (${k}_{6}$)	12.93	15.14	14.30	7.45	12.84	14.94	13.95	6.94
MSE (${k}_{7}$)	18.31	23.19	23.44	12.48	18.18	22.97	23.29	11.82
MSE (${k}_{8}$)	28.43	37.70	42.38	26.20	28.13	37.88	41.91	24.62
MSE (${k}_{9}$)	4.01	4.01	4.01	4.01	4.01	4.01	4.01	4.01
MSE (${k}_{10}$)	174.23	337.86	658.44	3331.87	172.08	328.74	640.68	3213.02
MSE (${k}_{11}$)	4.01	4.01	4.01	4.01	4.01	4.01	4.01	4.01
MSE (${k}_{12}$)	3.74	3.67	3.64	3.67	3.78	**3.72**	3.69	3.68
MSE (${k}_{13}$)	178.82	331.71	631.19	3128.52	178.02	324.28	616.99	3028.33
MSE (${k}_{14}$)	190.51	351.82	667.78	3303.17	188.90	343.34	650.95	3188.90
MSE (${k}_{15}$)	**3.59**	7.46	22.50	215.17	**3.57**	5.90	15.18	156.56
MSE (${k}_{16}$)	3.96	3.97	3.98	4.00	3.95	3.97	3.98	4.00

**Table 35:** Estimated MSEs for $\gamma =6$, $p=12$, and $n={50,\,100}$. Bold value indicates minimum MSE.

	*n* = 50				*n* = 100			
$\rho$	0.8	0.9	0.95	0.99	0.8	0.9	0.95	0.99
MSE (QLE)	630.35	1154.77	2511.53	13,610.04	290.59	542.26	1054.63	5190.68
MSE (${k}_{1}$)	488.65	905.89	1989.07	10,520.78	240.92	441.42	866.92	4226.55
MSE (${k}_{2}$)	380.93	696.68	1580.76	8653.98	184.13	350.71	689.16	3444.76
MSE (${k}_{3}$)	3.84	3.81	3.93	6.42	3.89	3.84	3.80	4.18
MSE (${k}_{4}$)	5.97	5.96	5.25	3.57	6.00	6.59	6.09	4.01
MSE (${k}_{5}$)	3.77	3.80	3.68	**3.49**	**3.26**	**3.30**	**3.31**	**3.40**
MSE (${k}_{6}$)	13.95	15.44	14.00	6.68	10.41	12.22	11.51	6.48
MSE (${k}_{7}$)	26.76	32.66	31.89	16.13	18.16	23.25	23.47	13.12
MSE (${k}_{8}$)	27.93	33.83	33.71	17.93	22.80	28.94	29.30	17.28
MSE (${k}_{9}$)	4.01	4.01	4.01	4.01	4.01	4.01	4.01	4.01
MSE (${k}_{10}$)	595.94	1119.24	2476.48	13,573.75	263.25	513.23	1025.26	5161.58
MSE (${k}_{11}$)	4.01	4.01	4.01	4.01	4.01	4.01	4.01	4.01
MSE (${k}_{12}$)	**3.60**	**3.59**	**3.62**	3.75	3.69	3.63	3.61	3.68
MSE (${k}_{13}$)	568.24	1040.40	2260.24	12,375.51	271.46	507.70	989.98	4879.23
MSE (${k}_{14}$)	614.78	1127.76	2441.50	13,302.14	286.66	534.99	1040.54	5122.12
MSE (${k}_{15}$)	25.90	81.30	248.60	1497.75	5.65	16.74	53.89	444.68
MSE (${k}_{16}$)	3.98	3.99	3.99	4.00	3.98	3.98	3.99	4.00

**Table 36:** Estimated MSEs for $\gamma =6$, $p=12$ and $n={150,\,200}$. Bold value indicates minimum MSE.

	*n* = 150				*n* = 200			
$\rho$	0.8	0.9	0.95	0.99	0.8	0.9	0.95	0.99
MSE (QLE)	279.84	517.18	1000.58	4825.58	274.33	504.66	973.03	4667.37
MSE (${k}_{1}$)	230.34	430.88	825.77	4002.55	224.08	419.49	814.61	3877.46
MSE (${k}_{2}$)	181.71	342.90	670.78	3282.32	179.63	337.16	657.56	3203.63
MSE (${k}_{3}$)	3.89	3.85	3.82	3.87	3.90	3.85	3.82	3.87
MSE (${k}_{4}$)	6.39	6.84	6.05	3.99	6.54	6.86	5.92	3.86
MSE (${k}_{5}$)	**3.26**	**3.22**	**3.20**	**3.39**	**3.36**	**3.27**	**3.21**	**3.39**
MSE (${k}_{6}$)	9.72	10.81	9.58	5.40	9.39	10.25	8.83	4.95
MSE (${k}_{7}$)	16.98	20.78	19.75	10.13	16.60	20.16	18.68	9.38
MSE (${k}_{8}$)	22.33	26.74	26.11	14.15	21.58	25.97	24.81	12.36
MSE (${k}_{9}$)	4.01	4.01	4.01	4.01	4.01	4.01	4.01	4.01
MSE (${k}_{10}$)	253.08	489.72	973.22	4798.11	247.59	477.22	945.07	4639.31
MSE (${k}_{11}$)	4.01	4.01	4.01	4.01	4.01	4.01	4.01	4.01
MSE (${k}_{12}$)	3.73	3.68	3.66	3.70	3.77	3.71	3.69	3.70
MSE (${k}_{13}$)	264.20	490.28	949.43	4584.04	260.23	480.16	926.76	4454.26
MSE (${k}_{14}$)	276.74	511.56	989.73	4773.68	271.53	499.63	963.38	4621.79
MSE (${k}_{15}$)	4.46	12.34	37.63	331.79	4.01	9.30	29.00	277.51
MSE (${k}_{16}$)	3.98	3.98	3.99	4.00	3.97	3.98	3.99	4.00

## Application: apprentice migration dataset

In this section, we explore the superiority of proposed estimators through a real-life example. We use this dataset^46^ to determine the performance of the proposed estimators. This real-life dataset is about apprentice migration between 1775 and 1799 to Edinburgh from Scotland. The dataset consists of a sample size of 33 ($$n=33)$$ with one explained variable and 4 predictors $$\left(p=4\right).$$ The explained variable $$y$$ denotes the apprentice and the independent variable $${x}_{1}$$ represents the distance, $${x}_{2}$$ represents the population, $${x}_{3}$$ represents the degree of urbanization and the $${x}_{4}$$ represents the direction from Edinburgh. The fitness of the QP distribution is determined by the estimated value of dispersion and the value of dispersion for this real-life data set was found to be 9651.93. By considering the dispersion value, it can be seen that this data of the concerned application is over-dispersed, so QPRM is more appropriate than the PRM.

As there are four predictors, there may be a chance of multicollinearity. To test multicollinearity among predictors, we consider the most popular criteria i.e. the condition index (CI). The CI is the square root of the ratio of minimum eigenvalue and maximum eigenvalue of the independent variables matrix. The CI value is 63.81 which is greater than 30, this shows that severe multicollinearity exists among the independent variables.

The QPRM estimates are obtained by using the QLE. The QLE can give better results if the predictors are uncorrelated. In this case, when the predictors are highly correlated with each other, so, the QLE does not provide good estimates. So, the QPRRE is considered to overcome the effect of multicollinearity. Table 37 shows the estimated coefficients and scalar estimated MSEs of the QLE and QPRRE under proposed RPEs. The QPRM estimates using the QLE and QPREE are obtained using Eqs. (6) and (10) respectively. The estimated scalar MSEs of the QLE and the QPRRE are obtained by using Eqs. (9) and (14) respectively. According to the results of Table 37, it is observed that the MSE of the QPRRE with RPEs is less than the MSE of the QLE. It means that the QPRRE performs well and gives the best results than the QLE. More specifically, the performance of the QPRRE with RPE ${k}_{7}$ is best as compared to the QPRRE with other RPEs and the QLE based on minimum MSE.

**Table 37:** Estimated regression coefficients and MSEs of the selected estimators in the Apprentice Migration Dataset. Bold value indicates minimum MSE.

Estimators	${\widehat{\beta }}_{0}$	${\widehat{\beta }}_{1}$	${\widehat{\beta }}_{2}$	${\widehat{\beta }}_{3}$	${\widehat{\beta }}_{4}$	MSEs	CV	PRESS
QLE	3.8472	− 0.0347	0.0211	− 0.0403	0.544	907.164	14.903	0.8262
${k}_{1}$	0.388	− 0.0194	0.0302	− 0.0127	1.0542	7.415	4.424	0.0163
${k}_{2}$	0.1069	− 0.0067	0.0308	0.0208	0.3942	1.64	2.391	0.0228
${k}_{3}$	0.0028	0.0069	0.031	0.0233	0.0101	4.76	1.086	0.0317
${k}_{4}$	0.0231	− 0.0003	0.0312	0.0332	0.0875	1.487	1.659	0.0327
${k}_{5}$	0.3708	− 0.019	0.0302	− 0.0116	1.0351	6.961	4.507	0.0156
${k}_{6}$	0.0241	− 0.0004	0.0312	0.0332	0.0914	1.474	1.692	0.0326
${k}_{7}$	0.0502	− 0.0027	0.031	0.03	0.1898	1.361	2.112	0.0293
${k}_{8}$	0.0305	− 0.001	0.0311	0.0326	0.1157	1.417	1.864	0.0318
${k}_{9}$	2.27E−16	8.55E−15	1.42E−14	5.82E−15	5.79E−16	15.1	1.004	0.1696
${k}_{10}$	0.0005	0.0108	0.0212	0.0097	0.0014	12.423	1.026	0.0450
${k}_{11}$	4.40E−13	1.66E−11	2.75E−11	1.13E−11	1.12E−12	15.1	1.000	0.1696
${k}_{12}$	0.0002	0.0058	0.01	0.0042	0.0005	14.332	1.016	0.0865
${k}_{13}$	3.812	− 0.0346	0.0212	− 0.0401	0.552	889.379	14.480	08,020
${k}_{14}$	3.8255	− 0.0346	0.0211	− 0.0402	0.5489	896.201	14.651	0.8112
${k}_{15}$	2.4101	− 0.0302	0.0249	− 0.0338	0.8664	325.778	5.152	0.2408
${k}_{16}$	0.0017	0.0091	0.0298	0.0189	0.0058	6.925	1.061	0.2376

In real datasets, sometimes the MSE criteria do not give good predictive performance of the estimators^47–49^. So, another model assessment criterion is recommended is the cross-validation (CV). The CV criteria are also known as the prediction sum of squares (PRESS/CV(1)) or a jackknife fit at given explanatory variables^50^. This criterion has also some limitations and different types^51^. The CV was considered by various authors for different models to assess the performance of their proposed estimators^47–49,52,53^. Here we consider the kfold CV and PRESS criterion for further evaluation of the proposed RPEs in the QPRRE. The procedure to compute the CV, we suggest to see the work of Akram et al.^52^ and Amin et al.^53^. While the PRESS is computed based on Pearson residuals for the QLE and QPRRE respectively as

$$PRESS\left(k=0\right)=\frac{1}{n}\sum_{i=1}^{n}\frac{{\chi }_{i}^{2}}{{\left(1-{h}_{ii}\right)}^{2}},$$

where ${\chi }_{i}=\frac{{y}_{i}-{\widehat{\mu }}_{i}}{\sqrt{\widehat{\gamma }{\widehat{\mu }}_{i}}}$, and ${h}_{ii}$ are the ith diagonal elements of the hat matrix computed for the QLE and

$$PRESS\left(k\right)=\frac{1}{n}\sum_{i=1}^{n}\frac{{\chi }_{ki}^{2}}{{\left(1-{h}_{kii}\right)}^{2}},$$,

where ${\chi }_{ki}=\frac{{y}_{i}-{\widehat{\mu }}_{ki}}{\sqrt{\widehat{\gamma }{\widehat{\mu }}_{ki}}}, {\widehat{\mu }}_{ki}$ is the predicted response of the QPRRE under different RPEs and ${h}_{kii}$ are the diagonal elements of the hat matrix obtained for the QPRRE. The estimated results for CV and PRESS for the QLE and QPRRE with all RPEs are given in Table 37. Based on CV results, it can be seen that the QPRRE with RPEs ${k}_{3}, {k}_{9}{-}{k}_{12}$ and ${k}_{16}$ gives a better performance as compared to the QPRRE with other RPEs as well as the QLE. When we look at the results of the PRESS criterion, we observed that the QPRRE with RPEs ${k}_{1}{-}{k}_{8}$ show a better performance than others. In view of simulation and application findings and we suggest the researchers to mitigate the effects of multicollinearity in QPRM, use the QPRRE with RPEs ${k}_{3}{-}{k}_{9}$, ${k}_{11}, {k}_{12}$ and ${k}_{16}$.

## Conclusion

When the dependent variable is in count form and over-dispersed, then the QPRM can be used for modeling such types of response variables. In this study, we proposed different RPEs for the QPRRE to minimize the problems of multicollinearity among the explanatory variables. To determine the superiority of the proposed ridge estimators, we conduct the simulation study under different parametric conditions such as different sample sizes, different numbers of predictor variables, different dispersion levels and different degrees of multicollinearity. Furthermore, the evaluation of the performance of proposed ridge estimators is done by analyzing the real-life dataset related to Apprentice migration data. According to the results of the simulation study and real-life dataset, it is observed that the QPRRE with some available and proposed RPEs outperforms as compared to the QLE in the presence of severe multicollinearity. The provided evidence showed that the QPRRE is a better estimation method than the QLE to combat the problem of multicollinearity among the explanatory variables for counts data with over-dispersion.

## Data Availability

All the datasets used in this paper are available from the corresponding author upon request.
